# Comparative Analysis of Four *Calypogeia* Species Revealed Unexpected Change in Evolutionarily-Stable Liverwort Mitogenomes

**DOI:** 10.3390/genes8120395

**Published:** 2017-12-19

**Authors:** Monika Ślipiko, Kamil Myszczyński, Katarzyna Buczkowska-Chmielewska, Alina Bączkiewicz, Monika Szczecińska, Jakub Sawicki

**Affiliations:** 1 Department of Botany and Nature Protection, University of Warmia and Mazury in Olsztyn, Plac Łódzki 1, 10-727 Olsztyn, Poland; kamil.myszczynski@gmail.com (K.M.); monika.szczecinska@uwm.edu.pl (M.S.); jakub.sawicki@uwm.edu.pl (J.S.); 2 Department of Biology, Institute of Experimental Biology, Adam Mickiewicz University in Poznań, Umultowska 89, 61-614 Poznań, Poland; androsac@amu.edu.pl (K.B.-C.); alinbacz@amu.edu.pl (A.B.)

**Keywords:** *Calypogeia*, editing sites, group I and II introns, intron loss, liverwort mitogenome, retroprocessing

## Abstract

Liverwort mitogenomes are considered to be evolutionarily stable. A comparative analysis of four *Calypogeia* species revealed differences compared to previously sequenced liverwort mitogenomes. Such differences involve unexpected structural changes in the two genes, *cox1* and *atp1*, which have lost three and two introns, respectively. The group I introns in the *cox1* gene are proposed to have been lost by two-step localized retroprocessing, whereas one-step retroprocessing could be responsible for the disappearance of the group II introns in the *atp1* gene. These cases represent the first identified losses of introns in mitogenomes of leafy liverworts (Jungermanniopsida) contrasting the stability of mitochondrial gene order with certain changes in the gene content and intron set in liverworts.

## 1. Introduction

Group I and group II introns, next to spliceosomal and transfer RNA (tRNA) introns, belong to four main types of introns divided on the basis of splicing mechanism [1]. Although two transesterification reactions are used by group I and group II introns for their splicing, the reaction mechanisms are different. As a result, group I introns are removed in a linear form, and some of them can circularize, whereas group II introns are released as a lariat [2]. Both discussed intron groups are also known as mobile elements. Their mobility is possible thanks to internal encoded enzymes, but the movement mechanism in each of the groups is different. Group I introns can proliferate by a DNA-mediated homing mechanism, where intron-encoded endonucleases play a key role. In contrast, retrohoming (RNA-mediated mechanism) is used by group II introns for propagation [3]. Internal encoded enzymes of this intron class, maturase, reverse transcriptase and endonuclease, enable retrohoming [4].

In addition to the differences in splicing and mobility mechanisms, group I and group II introns also have a unique structure. Group I introns have a characteristic RNA fold consisting of 10 elements denoted from P1 to P10 [5], while group II introns typically have a secondary structure consisting of six double-helical domains [2,6].

In plant mitochondrial genomes (chondriomes), group II introns occur more commonly than group I introns [1]. Group II introns are also present in archaebacteria, bacteria and plastids, but are absent in a nuclear genome, unless a sequence with this type of introns was transferred from mitochondrion to nucleus [7,8]. On the other hand, group I introns are common in nuclear ribosomic RNA (rRNA)-encoding genes, frequent in fungal mitochondria and are also present in plant organellar genomes, bacteria and viruses [1,9]. Intron contents of the mitochondrial genome in angiosperms are reported to be rather stable [10,11], as well as in hornworts, mosses and in the earliest land plants: liverworts. On the other hand, intron contents among these four distinct lineages are reported to be significantly different [11,12]. The tendency towards a stable number of introns in the mitogenome among bryophytes is in accordance with the hypothesis that their mitochondrial genomes are slowly evolving [13].

Retroprocessing is the most frequently reported mechanism for removal of introns [14,15,16,17,18]. According to this model, also known as a reverse transcriptase-mediated model (RT-mediated model), spliced mRNA is reverse-transcribed, and then, the emergent intronless complementary DNA (cDNA) is integrated into the genome by homologous recombination [14,15,16,17]. A loss of introns is thus associated with a loss of editing sites in the genome [18]. Under the standard type of this mechanism, the introns located at 3’ ends of genes are more likely to be deleted, because reverse transcriptase polymerizes the RNA template from 3’ ends to 5’ends of genes and frequently dissociates from the template without completing its rewriting [14,15,19]. Although reports of cDNA production in vivo are very rare [15,20,21], reverse transcription has been widely discussed in gene evolution [18], and a 3’ bias of intron loss has been reported in some organisms [16], which may indicate an RT-mediated model of intron loss. Intron losses from quillwort *Isoëtes engelmannii* [22], and gymnosperms are the strongest [23] evidence of retroprocessing in the plant mitochondrial genome.

Other possible mechanisms of intron loss include genomic deletion, exonization, horizontal gene transfer (HGT) and gene conversion. In the case of genomic deletion, introns are excised imprecisely [24], resulting in the removal of adjacent exonic sequences or retaining small fragments of introns, which are exonized [10]. This exonization may also involve an entire intron, which is no longer cut out of the transcript, but preserved in the mature mRNA and translated [25]. Disorders in intron cutting may be caused by creating alternative splice sites, which are the result of genomic insertions or point mutations in a DNA sequence. In this way, the splicing system includes new sequences (here introns) as exons or elongated existing exons [26]. Intron loss by deletion has been reported only in the case of *Petunia* [27], whereas exonization has never been evidenced in plant mitochondrial genome [10]. Intron loss can be also explained by HGT of an intron-less gene and the following gene conversion with an intron-including gene. The above mechanism in the plant mitogenome has only been observed in *Magnolia tripetala* to date [10].

The genus *Calypogeia* Raddi belongs to leafy liverworts (Jungermanniopsida) and consists of about 90 described species [28]. The most characteristic taxonomical feature of this genus is the presence of oil bodies and their color, shape and pattern of distribution in the leaf and underleaf [29]. This genus is the most widespread (but hardly cosmopolitan) genus of the family Calypogeiaceae. Although it includes species with wide Holarctic distribution (e.g., *Calypogeia suecica*, *Calypogeia sphagnicola*, *Calypogeia neesiana*), it also contains neotropical species occurring in Central and South America (e.g., *Calypogeia biapiculata*, *Calypogeia laxa*, *Calypogeia miquelii*) [30,31]. The family Calypogeiaceae is easily recognized, but species identification, due to the presence of environmentally-induced modifications and atypical forms and the frequent absence of sporophyte, can often lead to misidentification [32,33]. Some morphologically similar (cryptic) species are possible to distinguish using molecular markers [34].

Genomic work on liverworts mainly concerns simple (metzgeriid) and complex (marchantiid) thalloid liverworts, and there are still only a few sequenced mitogenomes [12,35,36,37] and plastomes [38,39,40,41,42,43].

In this paper, we introduce for the first time the mitochondrial genome structure of the genus *Calypogeia*. The four presented *Calypogeia* mitogenomes are the first sequenced mitochondrial genomes of leafy liverworts. The main goal of our research was to examine the stability of the mitochondrial genome structure among liverworts mentioned in the literature. Unexpectedly, we discovered a lack of introns in the genes *atp1* and *cox1*. It is the first case of intron loss within Jungermanniopsida leafy liverworts. We also discuss the possible mechanisms of intron disappearance.

## 2. Materials and Methods

### 2.1. Genome Sequencing, Assembly and Annotation

Total genomic liverwort DNA was extracted using ZR Plant/Seed DNA MiniPrepTM kit (Zymo Research Corp., Irvine, CA, USA) following the manufacturer’s recommendations. The DNA libraries of *Calypogeia integristipula*, *Calypogeia fissa* ssp. *fissa* and *Calypogeia suecica* were sequenced using the Illumina HiSeq 2000 platform (Illumina, San Diego, CA, USA) to generate 100 bp paired-end reads, and the DNA library of *Calypogeia arguta* was sequenced using HiSeqX (Illumina) to generate 150 bp paired-end reads (Table 1). After sequencing, the reads were cleaned by removing the adaptor sequences and low-quality reads with ambiguous sequences. Afterwards, the reads were mapped to the reference mitochondrial genome of *Aneura pinguis* using Geneious R8 software [44]. Then, gaps, in the obtained scaffold of the mitogenome sequence, were filled using an iterative fine-tuning step with minimum overlap and minimum overlap identity parameters set to 50 bp and 98%, respectively. The same genome assembly strategy was used for all Calypogeiaceae species analyzed in this study. The number of sequence reads and coverage depth for *C. arguta* deviates from other species since *C. arguta* has been deeply sequenced for nuclear genome research.

Genes were identified and annotated based on the closest known mitochondrial genomes of related species to the Calypogeiaceae, i.e., *Aneura mirabilis*, *A. pinguis*, *Pellia endiviifolia*, *Ptilidium pulcherrimum* and *Marchantia polymorpha*. Predictions were made using Geneious R8 software [44] and the BLAST tool [45]. Annotated sequences of the aforementioned Calypogeiaceae species were deposited in GenBank with the accession numbers specified in Table 1. A circular genome map, in order to visualize the structure of Calypogeiaceae mitogenome, was created on the basis of *C. fissa* ssp. *fissa* mitogenome using OGDraw software [46]. A MAUVE plot demonstrating the stability of the gene order in liverworts was made using Geneious R8 software [44] (Figure S1).

### 2.2. Prediction of RNA Editing Sites

Protein-coding sequences of *cox1* and *atp1* genes of four species (*A. pinguis*, *M. polymorpha*, *Pleurozia purpurea* and *Treubia lacunosa*) were downloaded from the NCBI (National Center for Biotechnology Information) database [47]. To predict editing sites and evaluate their editing rates within *cox1* and *atp1* genes, both the PREP-Mt [48] and PREPACT 2.0 [49] tools were used with a cutoff value of 0.8 with four downloaded and *Calypogeia fissa* ssp. *fissa*, *Tritomaria quinquedentata cox1* and *atp1* sequences.

## 3. Results and Discussion

### 3.1. Mitogenome Structure

**Figure 1:**
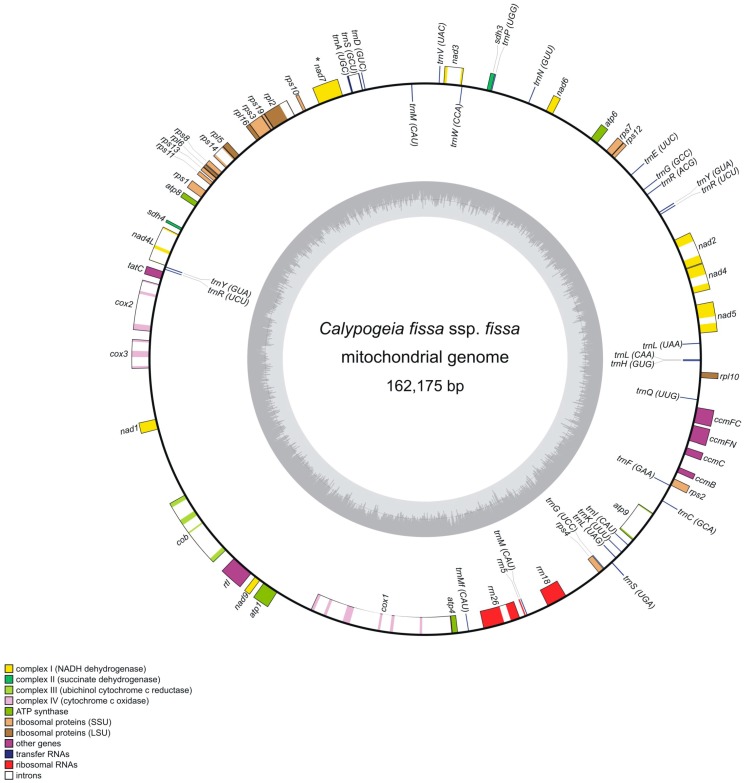
Gene map of the mitogenome of *Calypogeia fissa* ssp. *fissa*. Genes inside and outside the outer circle are transcribed in counterclockwise and clockwise directions, respectively. The genes are color-coded based on their function. The inner circle visualizes the G/C content. Pseudogenes have been marked with an asterisk.

The mitochondrial genome of *Calypogeia* is 159,061–163,057 bp in length (Table 1, Figure 1) and is slightly shorter than the closest related species with a known mitogenome structure: *P. purpurea* (168,526 bp) [36]. The length of the mitochondrial genome of *Calypogeia* is most similar to the mitogenome of *A. pinguis* (164,989 bp) [37]. The two other known mitochondrial genomes of the liverwort species, *T. lacunosa* [12] and *M. polymorpha* [38], differ more and are composed of 151,983 and 186,609 bp, respectively. The GC content in the studied genome (45%) is similar to other liverworts (42–45%) [12,36].

Seventy genes have been identified in the *Calypogeia* mitogenome: 42 protein-coding genes 25 tRNAs and three rRNAs (Table 2). Chondriomes of liverworts, similar to moss and hornwort mitogenomes, are reported to be rather static in gene content and order [12,13,50] and even with respect to pseudogene contents and retroposed pseudogene pieces [12]. The gene order in *Calypogeia* mitogenome is identical to the four aforementioned liverwort mitogenomes, although the gene content is slightly different between them. Mitogenomes of *M. polymorpha*, *P. purpurea*, *A. pinguis* and *Calypogeia* are very similar in gene composition. The differences occur mainly in the content of transfer RNA genes. *M. polymorpha* has only one copy of the *trnRucu*, but contains two more tRNA genes: *trnRucg* and *trnTggu*. One copy of the *trnRucu* has probably given rise to *trnRucg* [12]. The *trnT* gene is a part of the *trnA*-*trnT*-*nad7* gene cluster, whose different forms were identified by Wahrmund et al. [51] in liverwort mitogenome evolution. In leafy (jungermanniid) liverworts and in simple thalloid (metzgeriid), *trnT* is lacking between *trnA* and *nad7*, whereas in *Blasia pusilla*, representing a sister lineage to all other complex thalloid (marchantiid) liverworts, this gene occurs in conserved *Chara*-like version. The *trnT* gene is also present in *M. polymorpha* and other complex thalloid (marchantiid) liverworts, but its sequence is inverted compared to *Blasia*. Furthermore, in the *A. pinguis* mitochondrial genome, there is only one copy of the *trnYgua* in contrast to other liverwort mitogenomes [36]. Another difference concerns the *rtl* gene. In all aforementioned mitogenomes, this gene is functional with nucleotide sequence similarity > 84%, whereas in *P. purpurea*, it may be a pseudogene because of the high level of sequence divergence and several indels in the open reading frame [36]. However, a big part of the reading frame in *rtl* is intact, so this gene in *P. purpurea* may be still functional. The other dissimilarities occur between mitogenomes of the above four species and *T. lacunosa*. In the mitochondrial genome of the latter, either some genes of the cytochrome c biogenesis (*ccmC*, *ccmFN*) are missing or some of them are pseudogenized (*ccmB*, *ccmFC*). Another conspicuous dissimilarity concerns the *nad7* gene, which is only functional in *T. lacunosa* [12]. In most hornworts and liverworts, this gene is missing or occurs as a pseudogene with a degenerated structure [52,53,54], which is reflected in *Calypogeia* mitogenome and in the other sequenced mitochondrial genomes of liverworts. The only liverwort species with functional *nad7* are *Treubia* and *Haplomitrium* [52] belonging to Haplomitriopsida, a sister clade to the rest of the liverworts: Marchantiopsida and Jungermanniopsida [55] with the inactive *nad7* gene.

Ninety-four spacers and 24 introns (Table 3) have been found across the entire mitochondrial genome. The different length (1000–1300 bp) of the *nad5*-*nad4* spacer, containing the inverted sequence of the second *cob* intron in *M. polymorpha*, was recognized in different liverwort groups. Almost the entire *cob* intron sequence is inserted in the *nad5*-*nad4* spacer in marchantiid, while an internal region of this intron sequence copy is deleted among metzgeriid and jungermanniid taxa [56]. The above findings are also supported by the current study, because the *nad5*-*nad4* spacer in *Calypogeia* has a structure typical of jungermanniid liverworts.

**Figure 2:**
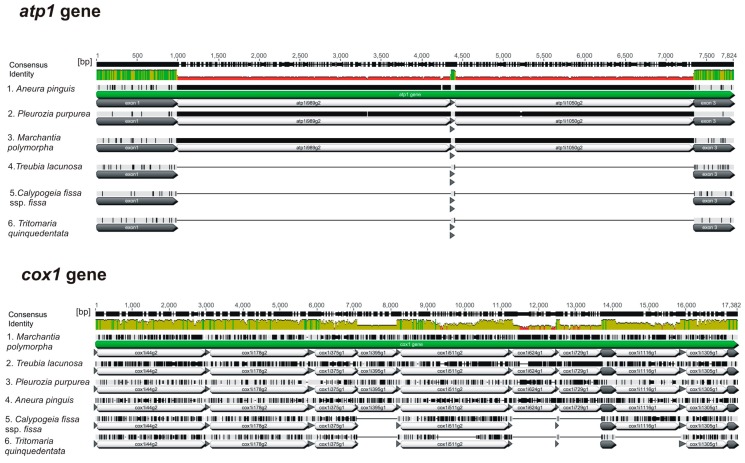
Comparison of the *atp1* and *cox1* gene structures of different liverwort species. Lost introns are marked with lines.

In the *Calypogeia* mitogenome, 22 introns (Table 3) are located in protein-coding genes, one in the *rrn26* gene and one in the *trnS* gene. Seven genes (*nad2*, *nad3*, *nad4*, *nad5*, *rpl2*, *rps14* and *atp9*) contain one intron. The genes *cox2*, *cox3* and *nad4L* have two introns, whereas the coding sequence of the *cob* gene is divided into three introns. The largest number of introns is localized in the *cox1* gene. However, surprisingly, only six introns occur in *Calypogeia*, whereas nine introns exist in the *cox1* gene of thalloid liverworts sequenced to date [12,35,36,37]. The *cox1* gene of *Calypogeia* lacks the cox1i395g1, cox1i624g1 and cox1i729g1 introns. The *atp1* gene has also lost two introns (atp1i989g2 and atp1i1050g2) and become in *Calypogeia* intronless (Figure 2). The CDS (protein-coding sequence) structure of both genes has not been affected. The intron set among species within each of the three major lineages of bryophytes is reported to vary slightly [12]. The intron number in the previously sequenced liverwort mitogenomes ranges from 28 in *T. lacunosa* to 30 in *M. polymorpha*, not including introns in pseudogenes. In liverworts, three cases of changes in the intron number have been detected to date (marked with numbers (1)–(3)). The rrn18i1065gII intron present in *M. polymorpha* is lacking in *P. purpurea* [35,36], *A. pinguis* [37] and in the examined genus *Calypogeia* (1). The intron set of *T. lacunosa* differs most compared to the other liverworts. Apart from the absence of the mentioned rrn18i1065gII intron (1), it lacks one intron in the *nad4L* gene (2) and, as in *Calypogeia*, two introns of the *atp1* gene (3) [12].

### 3.2. Possible Mechanisms of Intron Losses in Two Genes of Calypogeia

Among the currently sequenced liverwort mitogenomes, the *Calypogeia* mitochondrial genomes contain the fewest introns (24 introns). Three group I introns of the *cox1* gene and two group II introns in *atp1* are missing. Why have they disappeared? Deletion, as one of the intron loss mechanisms, can be ruled out. Introns in *Calypogeia* are precisely removed, and there are no intron fragments left or a deletion of small adjoining exon pieces. Exonization can also be rejected, because the introns physically disappeared from the *cox1* gene and the exon structures in both *atp1* and *cox1* genes remain intact. Furthermore, because of the large size of these introns (over 1000 bp), it would be unlikely [10]. Horizontal gene transfer and gene conversion also seem improbable since no chimeric structure in any place of the genes was noted. However, all *cox1* and *atp1* exons among liverworts are so similar, that even if horizontal transport and gene conversion took place, it would be hard to notice it.

**Figure 3:**
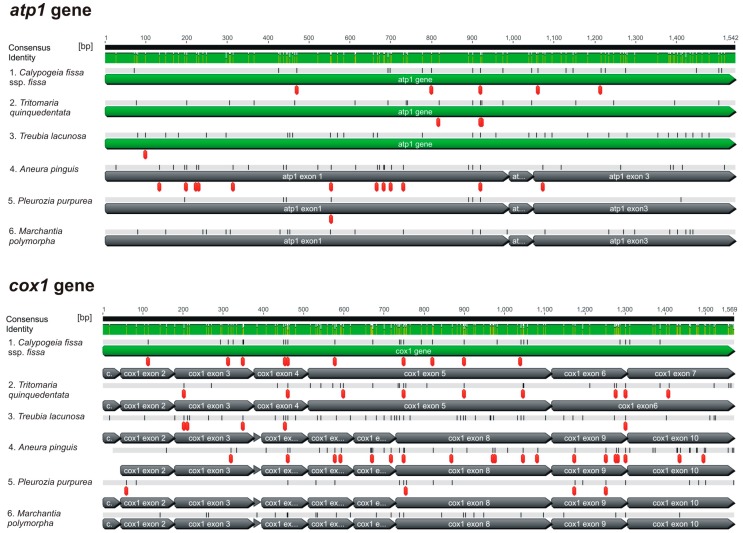
Occurrence of editing sites in *atp1* and *cox1* genes of different liverwort species (marked in red).

Taking into account the precision in the intron cut [10], the most probable mechanism of intron losses in *Calypogeia* genes is retroprocessing. However, this process in the *cox1* gene must have occurred at least twice, because non-intronic fragments are located in two places of the gene and are separated by a group II intron (Figure 2). This is the so-called localized retroprocessing, affecting only a part of the native gene [10,18]. Retroprocessing usually involves the removal of the adjacent edited sites, together with introns [18,22,59,60,61]. Computational analysis revealed 10 RNA editing sites in the *cox1* gene and five in the *atp1* gene of *Calypogeia* (Figure 3). Despite the intron deletions, nearby nucleotide positions that require editing remain. While the remaining two editing sites in the fourth exon of the *cox1* gene can be explained by embedding a cDNA fragment into a region between, but not including, the editing sites [18], the maintenance of edited sites in the fifth exon cannot be explained by this mechanism, because positions needing editing occur in the middle of this exon. Similarly, the loss of introns in the *atp1* gene does not seem to be related to the disappearance of the editing sites, because editing sites occur very close to the previous intron-exon boundary. Perhaps post-transcriptional modification of RNA in *Calypogeia* takes place in two successive stages: splicing and editing. Perhaps immediately after splicing, but before editing, RNA is reverse transcribed, and then, a partially-processed cDNA fragment undergoes conversion with the native intron-bearing gene. As a result, introns are removed, but editing sites remain [10,18]. The loss of introns without concurrent loss of flanking editing sites has recently been reported in ferns [62]. Edited sites from the intron-missing genes of *Calypogeia* may also not be removed because they are crucial for the excision of the remaining introns [10]. On the other hand, the introns can also play an important role in the correct splicing of the other ones [63]. Perhaps this is why the cox1i511g2 intron in the *cox1* gene of *Calypogeia* is preserved.

Apart from the precise intron excision, stronger evidence of retroprocessing is the loss of introns at the 3 ‘end of the gene. However, in the gene *cox1* of *Calypogeia*, introns have been lost in the center of the gene. A similar pattern of intron disappearance was observed by Nielsen et al. [64] who suggested that this may be caused either by a mutational mechanism (e.g., reverse transcription primed internally) or selective pressure to maintain introns near the 5′ and 3′ ends of genes. In *T. quinquedentata*, whose *cox1* gene also lacks the cox1i395gI, cox1i624gI and cox1i729gI introns, a loss of the cox1i1116g1 intron (3′-biased intron losses) was observed [65] (Figure 2), but the number and location of editing sites was very similar to those in *cox1* of *Calypogeia* (Figure 3). Thus, despite a visible lack of intron loss bias towards the 3’ end of the *cox1* gene in *Calypogeia* and the remaining editing sites, it is very likely that retroprocessing takes place in this gene.

## 4. Conclusions

In summary, the structure of the *Calypogeia* mitogenome is in line with reports of the stability of mitochondrial genomes in bryophyte lineages. The gene order is identical to other liverworts, while gene content fits the patterns that emerged in liverwort evolution. Dissimilarities in gene content among liverwort mitogenomes occur with regard to tRNA genes and protein-coding genes such as *rtl*, *nad7*, *ccmB* and *ccmFC* that are either functional or pseudogenized. Furthermore, *Calypogeia* species, like other sequenced liverworts, have functional *ccmC* and *ccmFN* genes, that are absent in *T. lacunosa*. The most unexpected difference occurs in the intron set. The *cox1* gene lacks three introns and *atp1* two. This is the first notification of intron losses in Jungermanniopsida leafy liverworts, although the disappearance of introns in *atp1* has already been reported in *T. lacunosa*. The mechanism responsible for intron disappearance seems to be retroprocessing: one-step retroprocessing in *atp1* and two-step localized retroprocessing in the *cox1* gene. The above findings indicate that the gene order in liverwort mitogenomes is stable, but the gene and intron contents may vary.

## Figures and Tables

**Table 1:** Sample species, voucher specimens and sequencing results of species used in this study. Mean coverage calculated as follows: number of reads that mapped to the mitogenome x average read length (bp)/mitogenome length (bp).

Species	Voucher	Sequencing Results (Total Number of Reads)	Reads Mapped to the Mitogenome (Number of Reads)	Average Read Length (bp)	Mitogenome Length (bp)	Mitogenome Mean Coverage	GenBank Accession Number
*Calypogeia arguta*	United Kingdom, collection. DC1420	383,522,854	2,665,062	147.9	159,061	2478.0	MF401630
*Calypogeia integristipula*	SE Poland, Bieszczady Mts, W slope of Mt Rozsypaniec Wołosacki, 1214 m, collection POZW 41928	19,604,000	69,739	99.6	163,057	42.6	MF401629
*Calypogeia fissa* ssp. *fissa*	W Poland, Lubuskie Province, Biecz forestry, collection POZW 42306	22,601,000	123,620	99.8	162,175	76.1	MF401632
*Calypogeia suecica*	SE Poland, Beskid Sądecki Mts, Potok Czarny stream, 717 m, collection POZW 42366	12,862,000	25,963	99.8	161,960	16.0	MF401631

**Table 2:** Gene contents in liverwort mitochondrial genomes. The black circle or “ψ” indicate the presence of a functional gene or a pseudogene, respectively. The white square indicates a gene lacking. Two black circles indicate the presence of a duplicated copy. ^a^ The gene *rtl* may be pseudogenized [36].

Gene/Species	*Treubia lacunosa*	*Marchantia polymorpha*	*Pleurozia purpurea*	*Aneura pinguis*	*Calypogeia fissa* ssp. *fissa*	*Tritomaria quinquedentata*
*atp1*	●	●	●	●	●	●
*atp4*	●	●	●	●	●	●
*atp6*	●	●	●	●	●	●
*atp8*	●	●	●	●	●	●
*atp9*	●	●	●	●	●	●
*ccmB*	ψ	●	●	●	●	●
*ccmC*	□	●	●	●	●	●
*ccmFC*	ψ	●	●	●	●	●
*ccmFN*	□	●	●	●	●	●
*cob*	●	●	●	●	●	●
*cox1*	●	●	●	●	●	●
*cox2*	●	●	●	●	●	●
*cox3*	●	●	●	●	●	●
*nad1*	●	●	●	●	●	●
*nad2*	●	●	●	●	●	●
*nad3*	●	●	●	●	●	●
*nad4*	●	●	●	●	●	●
*nad4L*	●	●	●	●	●	●
*nad5*	●	●	●	●	●	●
*nad6*	●	●	●	●	●	●
*nad7*	●	ψ	ψ	ψ	ψ	ψ
*nad9*	●	●	●	●	●	●
*rpl2*	●	●	●	●	●	●
*rpl5*	●	●	●	●	●	●
*rpl6*	●	●	●	●	●	●
*rpl10*	●	●	●	●	●	●
*rpl16*	●	●	●	●	●	●
*rps1*	●	●	●	●	●	●
*rps2*	●	●	●	●	●	●
*rps3*	●	●	●	●	●	●
*rps4*	●	●	●	●	●	●
*rps7*	●	●	●	●	●	●
*rps8*	●	●	●	●	●	●
*rps10*	●	●	●	●	●	●
*rps11*	●	●	●	●	●	●
*rps12*	●	●	●	●	●	●
*rps13*	●	●	●	●	●	●
*rps14*	●	●	●	●	●	●
*rps19*	●	●	●	●	●	●
*rrn5*	●	●	●	●	●	●
*rrn18*	●	●	●	●	●	●
*rrn26*	●	●	●	●	●	●
*rtl*	●	●	● **^a^**	●	●	●
*sdh3*	●	●	●	●	●	●
*sdh4*	●	●	●	●	●	●
*tatC*	●	●	●	●	●	●
*trnAugc*	●	●	●	●	●	●
*trnCgca*	●	●	●	●	●	●
*trnDguc*	●	●	●	●	●	●
*trnEuuc*	●	●	●	●	●	●
*trnFgaa*	●	●	●	●	●	●
*trnGgcc*	●	●	●	●	●	●
*trnGucc*	●	●	●	●	●	●
*trnHgug*	●	●	●	●	●	●
*trnIcau*	●	●	●	●	●	●
*trnKuuu*	●	●	●	●	●	●
*trnLcaa*	●	●	●	●	●	●
*trnLuaa*	●	●	●	●	●	●
*trnLuag*	●	●	●	●	●	●
*trnMcau*	●	●	●	●	●	●
*trnMfcau*	●	●●	●●	●●	●	●
*trnNguu*	●	●	●	●	●	●
*trnPugg*	●	●	●	●	●	●
*trnQuug*	●	●	●	●	●	●
*trnRacg*	●	●	●	●	●	●
*trnRucg*	□	●	□	□	□	□
*trnRucu*	●●	●	●●	●●	●●	●●
*trnSgcu*	●	●	●	●	●	●
*trnSuga*	●	●	●	●	●	●
*trnTggu*	□	●	□	□	□	□
*trnVuac*	●	●	●	●	●	●
*trnWcca*	●	●	●	●	●	●
*trnYgua*	●●	●●	●●	●●	●●	●●

ATP synthase subunits: *atp1*, atp4, *atp6*, *atp8*, *atp9*; Cytochrome c biogenesis subunits: *ccmB*, *ccmC*, *ccmFC*, *ccmFN*; Cytochrome c reductase subunits: *cob*; Cytochrome c oxidase subunits: *cox1*, *cox2*, *cox3*; NADH dehydrogenase subunits: *nad1*, *nad2*, *nad3*, *nad4*, *nad4L*, *nad5*, *nad6*, *nad7*, *nad9*; Large ribosomal protein units: *rpl2*, *rpl5*, *rpl6*, *rpl10*, *rpl16*; Small ribosomal protein units: *rps1*, *rps2*, *rps3*, *rps4*, *rps7*, *rps8*, *rps10*, *rps11*, *rps12*, *rps13*, *rps14*, *rps19*; Ribosomal RNAs: *rrn5*, *rrn18*, *rrn26*; Reverse transcriptase: *rtl*; Succinate dehydrogenase subunits: *sdh3*, *sdh4*; Twin arginine subunit c: *tatC*; Transfer RNAs: *trnAugc*, *trnCgca*, *trnDguc*, *trnEuuc*, *trnFgaa*, *trnGgcc*, *trnGucc*, *trnHgug*, *trnIcau*, *trnKuuu*, *trnLcaa*, *trnLuaa*, *trnLuag*, *trnMcau*, *trnMfcau*, *trnNguu*, *trnPugg*, *trnQuug*, *trnRacg*, *trnRucg*, *trnRucu*, *trnSgcu*, *trnSuga*, *trnTggu*, *trnVuac*, *trnWcca*, *trnYgua*.

**Table 3:** Intron contents in liverwort mitochondrial genomes. The black circle indicates the presence of an intron. The white square indicates an intron lacking. Intron nomenclature follows Dombrovska and Qiu [57] and Knoop [58].

Intron/Species	*Treubia lacunosa*	*Marchantia polymorpha*	*Pleurozia purpurea*	*Aneura pinguis*	*Calypogeia fissa* ssp. *fissa*	*Tritomaria quinquedentata*
atp1i989g2	□	●	●	●	□	□
atp1i1050g2	□	●	●	●	□	□
atp9i87g2	●	●	●	●	●	●
cobi372g2	●	●	●	●	●	●
cobi783g2	●	●	●	●	●	●
cobi824g2	●	●	●	●	●	●
cox1i44g2	●	●	●	●	●	●
cox1i178g2	●	●	●	●	●	●
cox1i375g1	●	●	●	●	●	●
cox1i395g1	●	●	●	●	□	□
cox1i511g2	●	●	●	●	●	●
cox1i624g1	●	●	●	●	□	□
cox1i729g1	●	●	●	●	□	□
cox1i1116g1	●	●	●	●	●	□
cox1i1305g1	●	●	●	●	●	●
cox2i97g2	●	●	●	●	●	●
cox2i250g2	●	●	●	●	●	●
cox3i171g2	●	●	●	●	●	●
cox3i625g2	●	●	●	●	●	●
nad2i709g2	●	●	●	●	●	●
nad3i140g2	●	●	●	●	●	●
nad4i548g2	●	●	●	●	●	●
nad4Li100g2	●	●	●	●	●	●
nad4Li283g2	□	●	●	●	●	●
nad5i753g1	●	●	●	●	●	●
nad7i336g2	●	●	●	●	●	●
nad7i1113g2	●	●	●	●	●	●
rpl2i28g2	●	●	●	●	●	●
rps14i114g2	●	●	●	●	●	●
rrn18i1065g2	□	●	□	□	□	□
rrn26i827g2	●	●	●	●	●	●
trnSgcui43g2	●	●	●	●	●	●

## Supplementary Materials:

The following are available online at www.mdpi.com/2073-4425/8/12/395/s1. Figure S1: Mauve alignment of six mitogenomes of liverworts.
