# Supplementary material for: Comparative Analysis of Four Calypogeia Species Revealed Unexpected Change in Evolutionarily-Stable Liverwort Mitogenomes
Source: Genes (Basel). 2017 Dec 19;8(12):395. doi: 10.3390/genes8120395 (PMC5748713; doi:10.3390/genes8120395)
Supplement: Supplementary file 1 [file genes-08-00395-s001.pdf]

Supplementary material

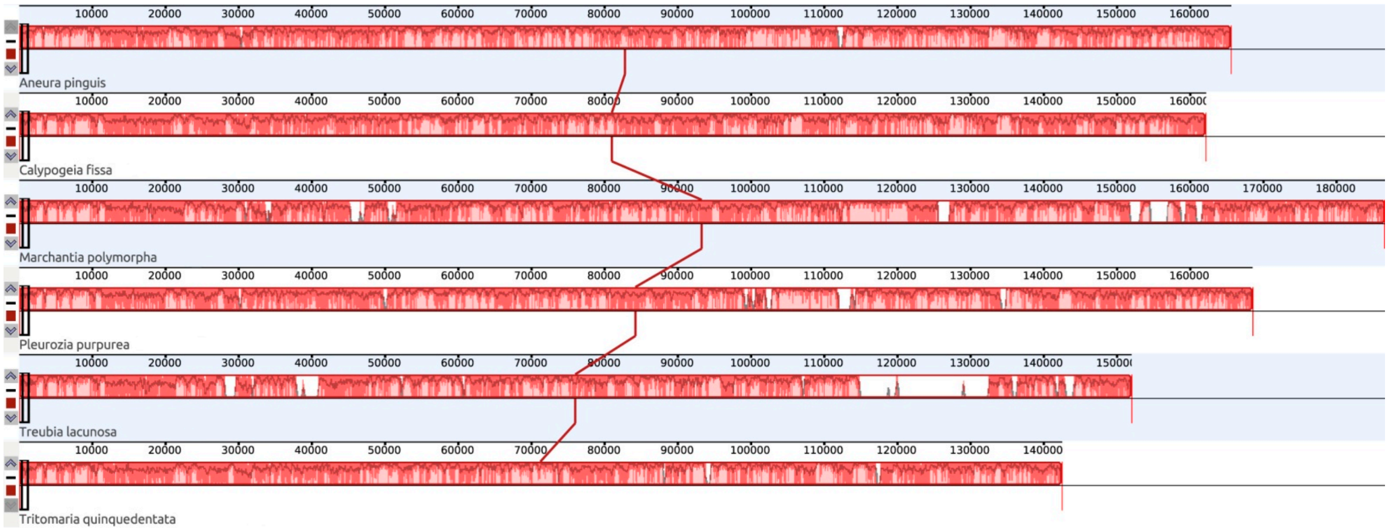

Figure S1. Mauve alignment of six mitogenomes of liverworts. Coloured regions represent high similarity of sequences while white spaces indicate low similarity and gaps. Due to the same gene order only one local linear block (LCB) has been discovered.
